# Selection with two alleles of X-linkage and its application to the fitness component analysis of *OdsH* in *Drosophila*

**DOI:** 10.1093/g3journal/jkae157

**Published:** 2024-07-13

**Authors:** Sha Sun, Chau-Ti Ting, Chung-I Wu

**Affiliations:** Department of Ecology and Evolution, University of Chicago, Chicago, IL 60637, USA; Department of Developmental and Cell Biology, University of California, Irvine, Irvine, CA 92697, USA; Department of Ecology and Evolution, University of Chicago, Chicago, IL 60637, USA; Institute of Ecology and Evolutionary Biology, National Taiwan University, Taipei 106, Taiwan; Department of Ecology and Evolution, University of Chicago, Chicago, IL 60637, USA; School of Life Sciences, Sun Yat-sen University, Guangzhou 510275, China

**Keywords:** X-linkage, selection, fitness components, two alleles, *OdsH*, sex bias

## Abstract

In organisms with the XY sex-determination system, there is an imbalance in the inheritance and transmission of the X chromosome between males and females. Unlike an autosomal allele, an X-linked recessive allele in a female will have phenotypic effects on its male counterpart. Thus, genes located on the X chromosome are of particular interest to researchers in molecular evolution and genetics. Here we present a model for selection with two alleles of X-linkage to understand fitness components associated with genes on the X chromosome. We apply this model to the fitness analysis of an X-linked gene, *OdsH* (16D), in the fruit fly *Drosophila melanogaster*. The function of *OdsH* is involved in sperm production and the gene is rapidly evolving under positive selection. Using site-directed gene targeting, we generated functional and defective *OdsH* variants tagged with the eye-color marker gene *white*. We compare the allele frequency changes of the two *OdsH* variants, each directly competing against a wild-type *OdsH* allele in concurrent but separate experimental populations. After 20 generations, the two genetically modified *OdsH* variants displayed a 40% difference in allele frequencies, with the functional *OdsH* variant demonstrating an advantage over the defective variant. Using maximum likelihood estimation, we determined the fitness components associated with the *OdsH* alleles in males and females. Our analysis revealed functional aspects of the fitness determinants associated with *OdsH*, and that sex-specific fertility and viability consequences both contribute to selection on an X-linked gene.

## Introduction

The X chromosome is present in both males and females of species with the XY sex-determination system. A daughter carries two copies of the X chromosome, one from each parent, whereas a son has only one X chromosome, inherited exclusively from his mother. The inheritance of X-linked genes differs from inheritance of autosomal genes in that father-to-son direct transmission of the X chromosome is prohibited: males receive the X chromosome only from their mothers and pass it on to their daughters only. Moreover, an X-linked recessive mutation is ineffective in heterozygous females, but causes mutant phenotypes in hemizygous males. As X-linked alleles are inherited unequally between sexes, natural selection on them must involve sex-specific life histories and can differ from selection on autosomal genes. There is cumulative evidence from whole-genome gene expression analyses that the X chromosome contains either over- or under-representations of sex-biased genes, whose activity is biased toward one sex over the other, in diverse organisms including *Drosophila*, mice, and humans (Wang *et al.* 2001; Lercher *et al.* 2003; Parisi *et al.* 2003; Ranz *et al.* 2003; Mueller *et al.* 2005; Zhang *et al.* 2010; Meisel *et al.* 2012). In X-linked genes, selection strength is related to the variation in gene functions between sexes. A sex-biased gene with X-linkage may indeed be subject to sex-specific selection (Vicoso and Charlesworth 2006). It is necessary to analyze the gene fitness effects in both sexes for the study of selection with X-linkage.

The *Odysseus*-site-homeobox (*OdsH*) gene in *Drosophila* is an X-linked protein-coding gene that is rapidly evolving and under positive selection (Ting *et al.* 1998). In *D. melanogaster*, expression of *OdsH* is male-biased and restricted to the testis, where *OdsH* appears to be dispensable, even though it significantly enhances sperm production in both young and older males (Sun *et al.* 2004; Cheng *et al.* 2012). The rapid evolution of *OdsH* gene sequence and its male sterility effect in hybrids of *D. simulans* and *D. mauritiana* can be attributed to the OdsH protein’s likely binding target—satellite DNA constituting heterochromatin with rapidly evolving sequences in the diverged *Drosophila* species (Bayes and Malik 2009). In *Drosophila*, the adaptive sequence changes and functional divergence of *OdsH* are also the result of gene duplication, which has given *OdsH* a new functional and evolutionary niche distinct from its paralog, *unc-4* (Ting *et al.* 2004; Nunes *et al.* 2023). As a hybrid male sterility gene, *OdsH*’s fitness consequences within a *Drosophila* species remain unclear, despite a deep understanding of its sequence evolution and function. The gene duplication that give rise to *OdsH* resulted in its function being dispensable for normal viability and fertility under laboratory conditions (Sun *et al.* 2004; Ting *et al.* 2004). In contrast, *OdsH* contributes significantly to species differentiation and evolution (Wu and Ting 2004; Maheshwari and Barbash 2011). We therefore examine whether the gain of *OdsH* function in a *Drosophila* species provides an overall fitness advantage.

A direct approach to assessing fitness effects and selection intensity would involve comparing fitness of two alleles over generations in a laboratory population (Prout 1969; Beckenbach 1983; Clark and Bundgaard 1984; Bastide *et al.* 2022). Generally, competition experiments are more appropriate for fitness analysis, and multi-generation experiments have been demonstrated to be more sensitive in detecting small differences in fitness as compared to single-generation experiments (Hartl and Jungen 1979; Wu *et al.* 1989; Fang *et al.* 2012). In multi-generation experiments, the fitness effect is manifested and accumulated over time and reflected in allele frequency changes across all generations in the population. A number of classical methods have been developed to determine the fitness components, i.e. viability and fertility, of autosomal alleles in experimental populations (Anderson 1969; Prout 1971a, 1971b; Beckenbach 1983; Clark and Bundgaard 1984). Using a mathematical model that addresses “Sex-Ratio” meiotic drivers, autosomal alleles and X chromosomes associated with sex-linked traits can also be analyzed (Wu 1983; Taylor and Jaenike 2002; Bastide *et al.* 2022). To analyze the fitness effects of an X-linked gene in the context of competing populations, we present here an experiment analysis combined with a mathematical model developed by Thomas Nagylaki; the model is an extension of the theory of “Selection with Two Alleles of X-linkage Based on Gamete Frequencies” (Nagylaki 1992). We apply the theoretical model to adult genotypic frequencies observed in competition experiments using a laboratory population setting.

The fitness component analysis of *OdsH* using the “Selection with Two Alleles of X-linkage” model is expected to determine whether *OdsH* function affects viability and fertility over continuous generations in male and female flies. Although *OdsH* has been shown to increase sperm production (Sun *et al.* 2004; Cheng *et al.* 2012), the fitness consequences of *OdsH* function remain unclear. In spite of the fact that *OdsH* expression has been shown to be male-biased and restricted to the testis (Sun *et al.* 2004; Ting *et al.* 2004), stage-specific RNA sequencing of the *D. melanogaster* transcriptome has revealed low levels of *OdsH* transcripts in embryonic, larval, and pupal stages of both males and females (Brown *et al.* 2014). Therefore, it is necessary to know whether and how *OdsH* may affect male and female viability over continuous generations. Furthermore, X-linked gene variants are hemizygous in males, whereas heterozygous or homozygous in females, requiring separate fitness component determinations for males and females.

## Selection with two alleles of X-linkage

On the basis of the same selection model with discrete non-overlapping generations previously described (Nagylaki 1992), we consider a single locus of X-linkage with two alleles $A_{1}$ and $A_{2}$ and no mutations. 
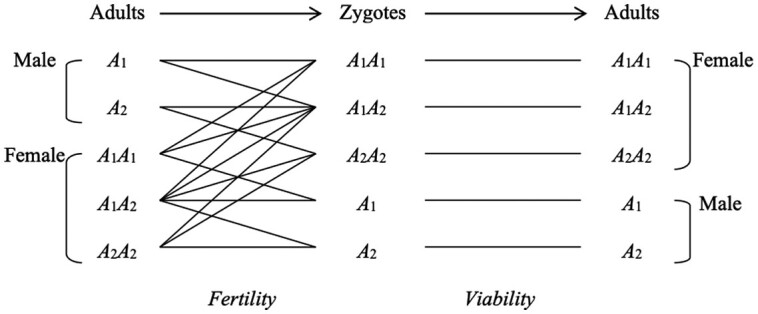


We use the following notations for adult frequencies in the population:


$$\begin{array}{rl} \;p_{1} & \;:\;\text{proportion of}\;A_{1}\;\text{males in the total males};\\ p_{2} & \;:\;\text{proportion of}\;A_{2}\;\text{males in the total males};\\ Q_{11} & \;:\;\text{proportion of}\;A_{1}A_{1}\;\;\text{females in the total females};\\ Q_{12}\;: & \;\text{proportion of}\;A_{1}A_{2}\;\;\text{females in the total females};\\ Q_{22} & \;:\;\text{proportion of}\;A_{2}A_{2}\;\;\text{females in the total females}. \end{array}$$

We define the following fertility effects:


$$\begin{array}{rl} a_{1} & \;:\;\text{average number of female zygotes born to an}\;A_{1}\;\text{male};\\ a_{2} & \;:\;\text{average number of female zygotes born to an}\;A_{2}\;\text{male};\\ b_{11} & \;:\;\text{average number of male zygotes born to an}\;A_{1}A_{1}\;\;\text{female};\\ b_{12} & \;:\;\text{average number of male zygotes born to an}\;A_{1}A_{2}\;\;\text{female};\\ b_{22} & \;:\;\text{average number of male zygotes born to an}\;A_{2}A_{2}\;\;\text{female};\\ c_{11} & \;:\;\text{average number of female zygotes born to an}\;A_{1}A_{1}\;\;\text{female};\\ c_{12} & \;:\;\text{average number of female zygotes born to an}\;A_{1}A_{2}\;\;\text{female};\\ c_{22} & \;:\;\text{average number of female zygotes born to an}\;A_{2}A_{2}\;\;\text{female}. \end{array}$$

Assuming sex-ratio is independent of genotype, then


$$\frac{b_{11}}{c_{11}}=\frac{b_{12}}{c_{12}}=\frac{b_{22}}{c_{22}}.$$

We define the viability effects:


$$\begin{array}{rl} u_{1} & \;:\;\;\text{probability an}\;A_{1}\;\text{male survives to reproductive age};\\ u_{2} & \;:\;\;\text{probability an}\;A_{2}\;\text{male survives to reproductive age};\\ v_{11} & \;:\;\;\text{probability an}\;A_{1}A_{1}\;\;\text{female survives to reproductive age};\\ v_{12} & \;:\;\;\text{probability an}\;A_{1}A_{2}\;\;\text{female survives to reproductive age};\\ v_{22} & \;:\;\;\text{probability an}\;A_{2}A_{2}\;\;\text{female survives to reproductive age}. \end{array}$$

We describe the ratios for genotypic frequencies and fitness (i.e. fertility and viability) effects:


$$\begin{array}{rl} \text{adult frequencies} & \;:\;R_{1}=\frac{\;p_{1}}{p_{2}},\;R_{2}=\frac{Q_{11}}{Q_{22}},\;R_{3}=\frac{Q_{22}}{Q_{12}};\\ \;\text{fertility effects} & \;:\;\alpha =\frac{a_{1}}{a_{2}},\;\beta =\frac{b_{11}}{b_{12}}=\frac{c_{11}}{c_{12}},\;\gamma =\frac{b_{22}}{b_{12}}=\frac{c_{22}}{c_{12}};\\ \text{viability effects} & \;:\;\mu =\frac{u_{1}}{u_{2}},\;\nu =\frac{v_{11}}{v_{12}},\;\epsilon =\frac{v_{22}}{v_{12}}. \end{array}$$

We shall suppose that *α*, *β*, *γ*, *μ*, *ν* , ϵ are constant.

The next generation adult frequency ratios can be derived as


(1) $$R_{1}^{\prime }=\frac{\;p_{1}^{\prime }}{p_{2}^{\prime }}=\frac{(Q_{11}b_{11}+(1/2)Q_{12}b_{12})u_{1}}{(Q_{22}b_{22}+(1/2)Q_{12}b_{12})u_{2}}=\frac{(2R_{2}\beta +1)\mu }{2R_{3}\gamma +1},$$

(2) $$\begin{array}{rl} R_{2}^{\prime } & =\;\frac{Q_{11}^{\prime }}{Q_{12}^{\prime }}\\ & =\;\frac{(p_{1}a_{1}Q_{11}c_{11}+(1/2)p_{1}a_{1}Q_{12}c_{12})v_{11}}{(p_{1}a_{1}Q_{22}c_{22}+p_{2}a_{2}Q_{11}c_{11}+(1/2)p_{1}a_{1}Q_{12}c_{12}+(1/2)p_{2}a_{2}Q_{12}c_{12})v_{12}}\\ & =\;\frac{(2R_{2}\beta +1)R_{1}\alpha \nu }{2R_{1}R_{3}\alpha \gamma +2R_{2}\beta +R_{1}\alpha +1}, \end{array}$$

(3) $$\begin{array}{rl} R_{3}^{\prime } & =\;\frac{Q_{22}^{\prime }}{Q_{12}^{\prime }}\\ & =\;\frac{(p_{2}a_{2}Q_{22}c_{22}+(1/2)p_{2}a_{2}Q_{12}c_{12})v_{22}}{(p_{1}a_{1}Q_{22}c_{22}+p_{2}a_{2}Q_{11}c_{11}+(1/2)p_{1}a_{1}Q_{12}c_{12}+(1/2)p_{2}a_{2}Q_{12}c_{12})v_{12}}\\ & =\;\frac{(2R_{3}\gamma +1)\epsilon }{2R_{1}R_{3}\alpha \gamma +2R_{2}\beta +R_{1}\alpha +1}. \end{array}$$

## Site-directed gene modification in *OdsH*

To investigate the fitness effects of an X-linked gene in *Drosophila*, we introduce specific gene modifications to perturb the gene function, and tag the gene with an easily traceable marker. Our previous study (Sun *et al.* 2004) used the site-directed “gene targeting” method (Rong and Golic 2000) to insert the eye-color marker gene *white* into the X-linked *OdsH* gene locus of *Drosophila melanogaster*.

**Fig. 1.**
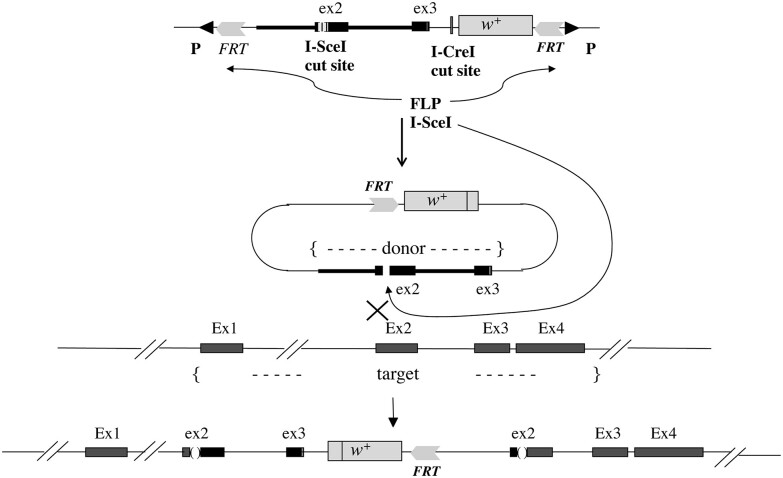
Gene targeting at *OdsH*. The targeting construct (top) contains the direct repeats of the recombination target (FRT) in a P-element, which carries a homologous genomic fragment of *OdsH* Exon 2 and partial Exon 3 (ex3) with the marker gene *white* ($w^{+}$). The constructed Exon 2 (ex2) contains a restriction enzyme recognition site for I-SceI. Heat-shock-induced FLP enzyme in the transgenic flies will cause the excision of the FRT-flanked donor DNA, followed by restriction digest induced by the I-SceI enzyme synthesized in these flies resulting in a double-strand break in ex2. In accordance with the double-strand DNA break repair mechanism, homologous recombination between the donor and the endogenous *OdsH* will result in the integration of the donor DNA into the *OdsH* locus with the endogenous Exon 1 (Ex1), Exon 2 (Ex2), Exon 3 (Ex3), and Exon 4 (Ex4). A mutation resulting from an imprecise integration could affect the gene structure and function of *OdsH*.

**Fig. 2.**
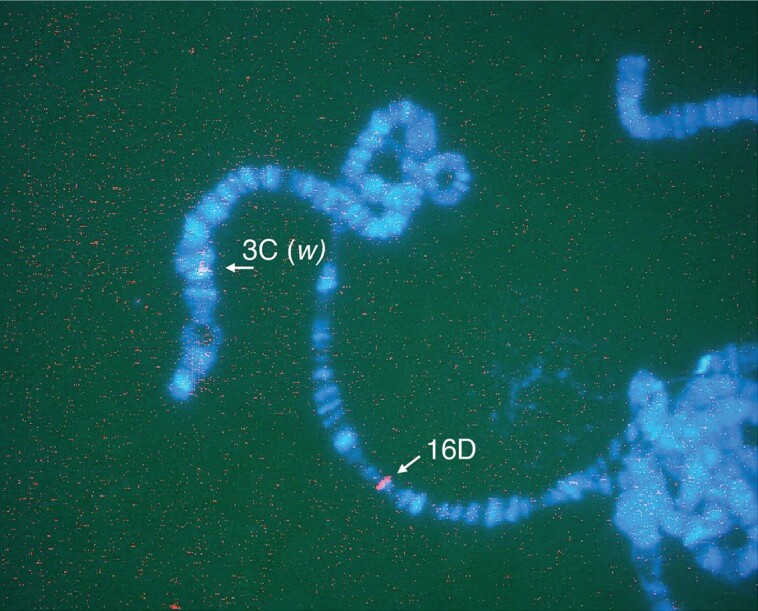
DNA fluorescent in situ hybridization (FISH) of a polytene chromosome. Chromosome X from a transgenic male fly with modified *OdsH* is identified by DNA FISH against the marker gene *white* in polytene chromosomes. A digoxigenin (DIG) labeled *white* DNA probe was detected with anti-DIG rhodamine conjugate (red) at cytological positions 3C (arrow corresponding to the endogenous *white*(*w*) gene locus) and 16D (arrow corresponding to the endogenous *OdsH* gene locus). Polytene chromosomes with compacted bands are visualized by DAPI staining (blue).

As shown in Fig. 1, we used a targeting construct that carried the 3.8 kb homologous genomic region of *OdsH* Exon 2 and partial Exon 3 to achieve a site-directed gene modification. The modified *OdsH* Exon 2 contained a restriction enzyme I-SceI cut-site. The entire targeting construct was introduced into the fly genome using P-element mediated transgenesis (Spradling *et al.* 1999; Rong and Golic 2000). Homologous recombination was induced by heat-shock activated Flp and I-SceI enzymes, leading to a double-strand break and the donor DNA integration at *OdsH* Exon 2. The gene targeting scheme requires double-strand DNA repair mechanisms, which could also result in frame-shifts and premature stop-codons in *OdsH*. In Fig. 2, we demonstrated that the *white* marker gene was integrated into the X chromosome at the *OdsH* locus. We also obtained a number of independent gene modification events in *OdsH*, establishing separate fly lines with modified *OdsH* gene variants.

**Fig. 3.**
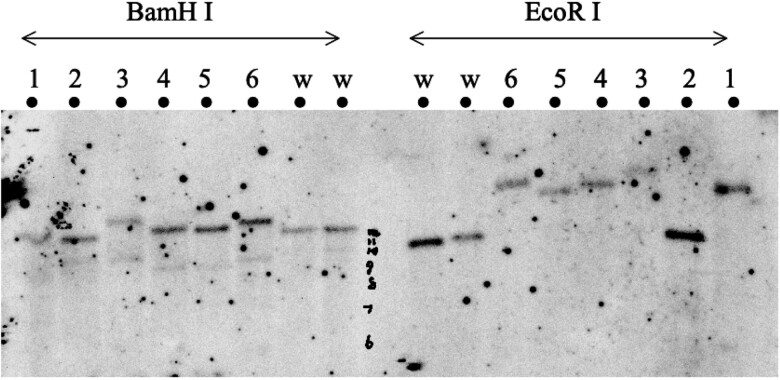
Southern blotting of the genomic DNA isolated from transgenic flies. Insertion of the donor DNA at *OdsH* is validated by genomic DNA size increase detected by a DIG labeled *OdsH* probe on genomic DNA digested by restriction enzyme *Eco*R I or *Bam*H I. Six *OdsH* transgenic lines (#1, #2, #3, #4, #5, and #6) from independent gene modification events were compared with two wild-type (w) control lines.

As shown in Fig. 2, an integration of the targeting construct at the endogenous *OdsH* gene was validated by detecting the *white* marker in the polytene chromosome band 16D. The genomic DNA change at the *OdsH* gene locus was confirmed by Southern blotting (Fig. 3). Except for #2, a mistargeting event that did not affect the *OdsH* gene structure, all five other events caused genomic DNA changes in *OdsH*, demonstrating various length increases in the genomic DNA as compared with the wild-type samples. We chose the #1 targeting event and named the modified allele ${OdsH}^{1}$.

**Fig. 4.**
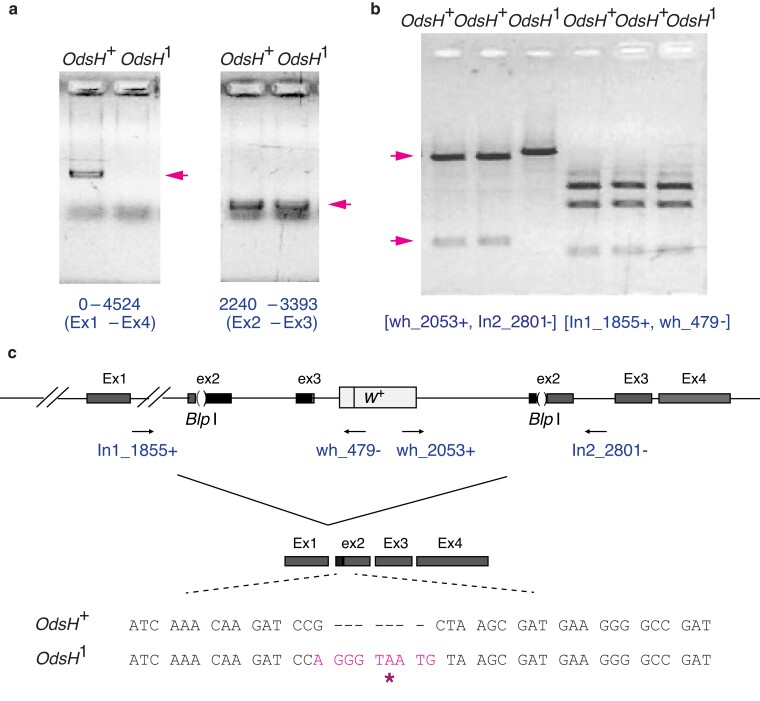
RNA reverse transcription (RT)-PCR and genomic long-template PCR on *OdsH* targeting lines. Left panel of a) shows the full-length transcript of *OdsH* (pink arrow) corresponding to nucleotide positions 0–4524 and Exon 1–Exon 4 (Ex1–Ex4) detected by RT-PCR in the ${OdsH}^{+}$ line; no full-length transcript is detected in the ${OdsH}^{1}$ line. Right panel of a) shows the short transcript of *OdsH* (pink arrow) corresponding to nucleotide positions 2240–3393 and Exon 2–Exon 3 (Ex2–Ex3) detected by RT-PCR in both ${OdsH}^{+}$ and ${OdsH}^{1}$ lines. b) The long-template PCR followed by *Blp*I digestion, resolving the genomic DNA structure at *OdsH* after gene targeting. The PCR amplicon obtained from the primer pair (wh 2053+, In2 2801−) can be digested completely by the restriction enzyme *Blp*I resulting in two fragments for the two ${OdsH}^{+}$ lines (pink arrows); however, the same amplicon for the ${OdsH}^{1}$ line cannot be digested by *Blp*I, resulting in one large DNA fragment. The PCR amplicon obtained from the primer pair (In1 1855+, wh 479−) can also be digested by *Blp*I, resulting in three DNA fragments for all three lines, the same for ${OdsH}^{+}$ and ${OdsH}^{1}$. As shown in c), the schematic structure of *OdsH* after gene targeting includes the endogenous Exon 1 (Ex1), the modified Exon 2 (ex2), partial Exon 3 (ex3), the *white* marker gene ($w^{+}$), the downstream modified Exon 2 (ex2), and the endogenous Exon 3 (Ex3) and Exon 4 (Ex4). The primers (arrows) used in the genomic long-template PCR are indicated with their corresponding locations to *OdsH* Intro 1 (In1 1855+), the *white* marker gene, (wh 479−) & (wh 2053+), and *OdsH* Intron 2 (In2 2801−). A full-length *OdsH* containing all four exons, shown as Ex1–ex2–Ex3–Ex4, is produced by splicing from Exon 1 to modified Exon 2 downstream of the *white* marker; whereas ${OdsH}^{+}$ has an Exon 2 coding sequence same as wild-type Exon 2, ${OdsH}^{1}$ has nucleotide insertion (pink) in the modified Exon 2, resulting in a stop codon (*) and frameshift.

As shown in Fig. 4a, ${OdsH}^{1}$ lacks the full-length transcript of *OdsH* but has a short transcript covering Exon 2 and partial Exon 3. The genomic structure at the target site was resolved by long-template PCR using *white* gene primers and *OdsH* intron primers (Fig. 4b and c). As *OdsH* contains an endogenous *Blp*I enzyme cut-site, restriction digestion by *Blp*I was used to determine whether Exon 2 integration was precise. As shown in Fig. 4, for the ${OdsH}^{+}$ lines, precise integration resulted in the restoration of both *Blp*I sites and successful digestion of the both PCR amplicons. By contrast, for the ${OdsH}^{1}$ line, one of the PCR amplicons failed to be digested by *Blp*I and appeared on the gel as an intact DNA fragment. In the ${OdsH}^{+}$ lines, a full-length *OdsH* transcript can be spliced while skipping the *white* marker gene and using the downstream modified Exon 2 and endogenous *OdsH* Exons 3 and 4 (Fig. 4c). Similar splicing patterns have also been observed in site-directed gene targeting procedures performed by others (D. Pauli and Y. S. Rong, personal communication). The precise integration and intact Exon 2 coding nucleotides in ${OdsH}^{+}$ were validated by DNA sequencing as shown in 4c, while an imprecise integration resulted in nucleotide insertions with a stop codon and frameshift mutation in ${OdsH}^{1}$. Such a premature stop codon can cause nonsense-mediated decay of the mRNA and transcript degradation (Lytle and Steitz 2004). Accordingly, ${OdsH}^{1}$ does not have a full-length transcript; the short transcript is the splicing product of *OdsH* exons upstream of the *white* marker, which include an intact Exon 2 and a partial Exon 3.

## Laboratory population for allelic competition

Our experimental system is *D. melanogaster* with site-directed *OdsH* modification, which allows us to apply the X-linked gene selection model and laboratory populations to understanding the fitness components associated with the *OdsH* gene function. In contrast to ${OdsH}^{+}$, which represents wild-type *OdsH* with full-length RNA, ${OdsH}^{1}$ produces only a truncated RNA, which represents a loss-of-function *OdsH* mutant. Despite the absence of a full-length RNA, ${OdsH}^{1}$ flies appear normal under laboratory conditions. In accordance with our understanding of *OdsH* in *D. melanogaster*, even the null-mutant ${OdsH}^{0}$ flies do not exhibit any observable defects in morphology, viability, or fertility unless sperm exhaustion conditions were applied, in which ${OdsH}^{0}$ significantly reduced male fertility (Sun *et al.* 2004; Cheng *et al.* 2012). The fitness effects of a rapidly evolving gene like *OdsH* can be challenging to infer. Unlike obvious phenotypic changes under normal laboratory conditions, fitness differences are not always apparent. Therefore, population experiments are necessary to reveal whether aspects of the gene function determine the fitness components—survival and/or reproductive advantages over multiple generations.

**Fig. 5.**
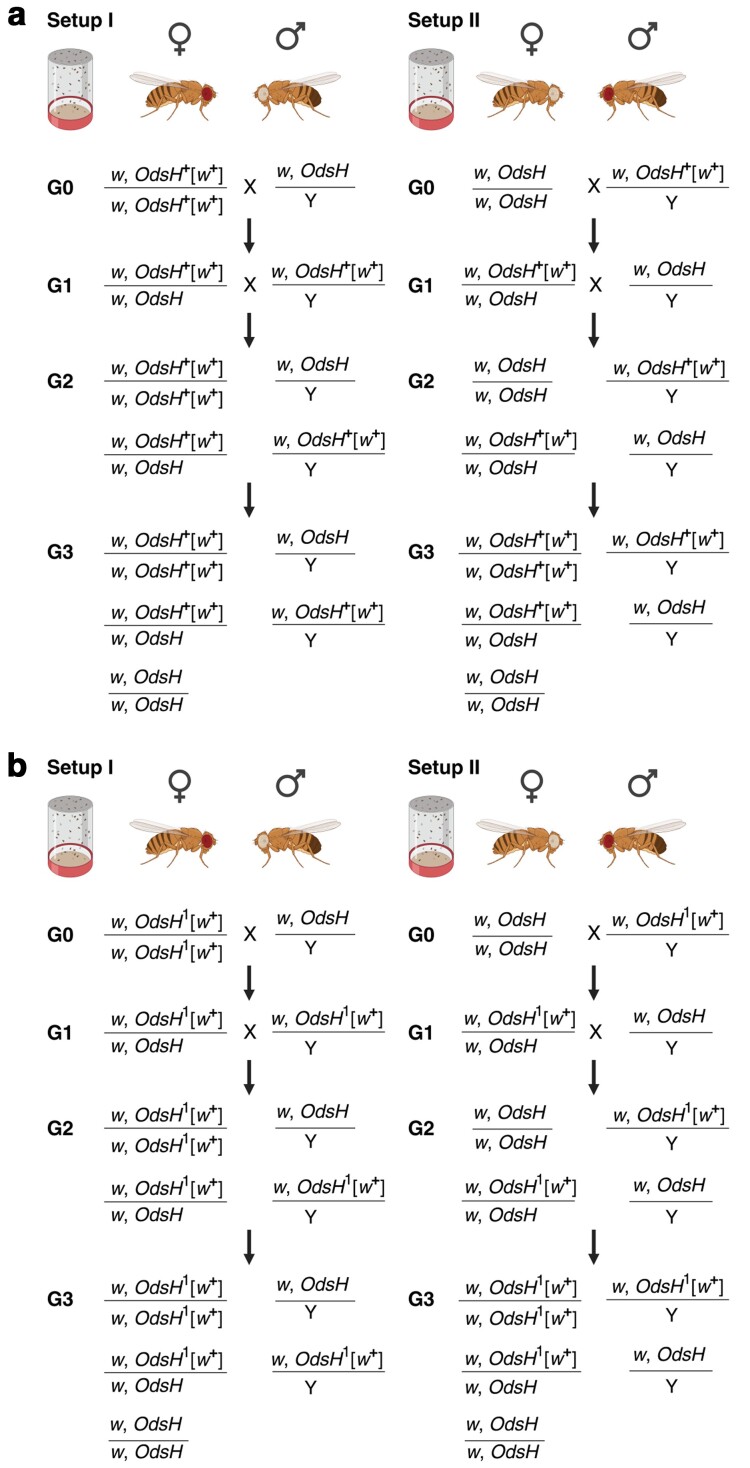
*Drosophila* cross for population setup. As shown in a), two reciprocal crosses were carried out to set up fly populations for competing ${OdsH}^{+}$ against the wild-type *OdsH*: “Setup I” starts with females of homozygous ${OdsH}^{+}$ carrying the *white* marker gene $w^{+}$ in the *white*-mutant *w* genetic background (flies are red-eyed) and males of wild-type *OdsH* in the *white*-mutant *w* background (flies are white-eyed); in parallel, “Setup II” starts with females of homozygous wild-type *OdsH* in the *white*-mutant *w* background (flies are red-eyed) and males of ${OdsH}^{+}$ carrying the *white* marker gene $w^{+}$ (flies are white-eyed). Every generation includes the indicated genotypes; G0 initiates the population; G1 is the starting generation, followed by G2 and G3. b) The same scheme with two reciprocal crosses to set up fly populations for competing ${OdsH}^{1}$ against the wild-type *OdsH*. ${OdsH}^{1}$ is associated with $w^{+}$ the same way as ${OdsH}^{+}$.

To examine the fitness effects of *OdsH*, two different alleles were introduced into a laboratory population for allelic competition (Fig. 5). In an otherwise white-eyed fly with *white*-mutant *w* genetic background, the marker gene *white*, $w^{+}$, provides an easily identifiable red eye-color trait. As a result of integrating $w^{+}$ into *OdsH*, both ${OdsH}^{+}$ and ${OdsH}^{1}$ alleles are tagged with $w^{+}$, but differ only in transcript length. As shown in Fig. 5, the fly cross scheme is used to set up laboratory populations competing two alleles, ${OdsH}^{+}$ (associated with $w^{+}$) and the wild-type *OdsH* (associated with *w*) (Fig. 5a); at the same time, ${OdsH}^{1}$ (with $w^{+}$) and *OdsH* (with *w*) are set up as two competing alleles in populations following the same scheme (Fig. 5b). For each setup at G0, vials of initiating cross containing three virgin females and three males were set up and these parental adult flies were discarded on day 8 after egg-laying; the eclosed progeny (∼200 flies collected from two vials) were transferred into a 3/4 pint milk bottle without anesthesia on day 14 to start the population at G1. After transfer, flies were allowed to mate and lay eggs in the bottle for 5 days before being collected and scored; the new generation eclosed for another 10 days, and the young adults (G2) were transferred to a fresh bottle without anesthesia to continue the population and produce the next generation (G3). The adult male and female flies were scored based on eye-color: males had white eyes (*w*) or red eyes ($w^{+}$); females had white eyes (*w*/*w*) or orange-red eyes (*w*/$w^{+}$ or $w^{+}$/$w^{+}$). The generations are discrete and non-overlapping because all the parent adult flies were collected before the next generation young eclosed.

Laboratory population studies of flies have been conducted using fly bottles of various sizes (Clark 1979; Beckenbach 1983; Sgro and Partridge 2000; Houle and Rowe 2003; Phillips *et al.* 2016; Bastide *et al.* 2022). Fly bottles take up less space than large population cages and allow parallel setups of multiple populations at the same time. In addition, fly bottles are easier to handle and provide a relatively controlled environment for populations over multiple generations. In each setup, two replicate populations were maintained in separate bottles, and we observed that there was no significant difference between the duplicate bottles. All population bottles were kept at 22–23 ° C with day and night cycles. The population size ranged from 500 to 800 flies per bottle, which is consistent with the average number of flies transferred during normal culture of the wild-type flies in a 3/4 pint bottle.

In contrast to allele frequencies for autosomal genes, which can be calculated by averaging genotypic frequencies in males and females, allele frequencies for X-linked genes must take into account the fact that females carry two copies and males carry one copy. Therefore, we have to consider X-linked genotypic frequencies separately in males and females. As illustrated in Fig. 5, the reciprocal crosses, setup I and setup II, initiate the allelic competition populations at G1 with the same *OdsH*(*w*) frequency in females (heterozygotes) at 0.5; however, the *OdsH*(*w*) frequency in males differs: 0 for setup I and 1 for setup II. Populations can have different outcomes based on different starting frequencies over multiple generations. With no selection, the allele frequency for *OdsH*(*w*) is expected to be 1/3 for setup I and 2/3 for setup II.

**Fig. 6.**
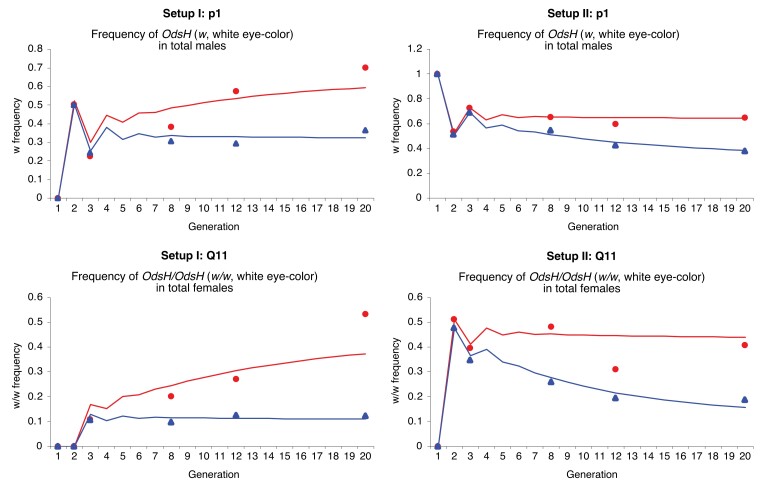
Genotypic frequencies of wild-type *OdsH* (represented by *w*, white eye-color) in adult males and wild-type *OdsH*/*OdsH* (represented by *w*/*w*, white eye-color) in adult females over 20 generations of allelic competition in laboratory populations. As described in Fig. 5a, “setup I” (left panels) starts with females of homozygous ${OdsH}^{+}$ ($w^{+}$) and males of wild-type *OdsH* (*w*) at G0; therefore, starting in generation 1, the frequency for wild-type *OdsH* in adult males (p1) is 0 and the frequency for wild-type *OdsH*/*OdsH* in females (Q11) is also 0; the theoretical model for neutrality predicts p1 to be 0.333 and Q11 to be 0.111 by generation 20. By contrast, “setup II” (right panels) starts with females of homozygous wild-type *OdsH* (*w*) and males of ${OdsH}^{+}$ ($w^{+}$); therefore, starting in generation 1, the frequency for wild-type *OdsH* in adult males (p1) is 1 and the frequency for wild-type *OdsH*/*OdsH* in females (Q11) is 0; the theoretical model for neutrality predicts p1 to be 0.667 and Q11 to be 0.444 by generation 20. The generation numbers 1, 2, 3 correspond to G1, G2, G3 as illustrated in Fig. 5. Blue triangles are experimental data following the competition between ${OdsH}^{+}$ [$w^{+}$] and *OdsH* [*w*]; red circles are experimental data following the competition between ${OdsH}^{1}$ [$w^{+}$] and *OdsH* [*w*]. Blue lines and red lines are corresponding best-fit models for adult genotypic frequencies derived as equations (7) and (8). Frequency values are included in Supplementary Table S1.

It was our goal to uncover differences in fitness between ${OdsH}^{+}$ and ${OdsH}^{1}$, both carry the $w^{+}$ marker through the same gene targeting procedures, but they differ in the length of the *OdsH* transcript that is produced. These two modified *OdsH* variants show no visible phenotypic differences, so it was not feasible to set them as two competing alleles in the same population. A concurrent setup of ${OdsH}^{+}$ and ${OdsH}^{1}$, each competing against the wild-type *OdsH* allele in a separate population, would allow us to examine the allele frequency changes and fitness components of ${OdsH}^{+}$ and ${OdsH}^{1}$ individually against the wild-type *OdsH* allele, as well as to determine the differences between ${OdsH}^{+}$ and ${OdsH}^{1}$. The fitness differences resolved between ${OdsH}^{+}$ and ${OdsH}^{1}$ would be due to genetic differences in the *OdsH* RNA (full-length vs partial transcript) that indicate functional vs nonfunctional *OdsH*. The tight linkage of the $w^{+}$ marker for the modified *OdsH* variants facilitates the tracking of allele frequencies in competing with the wild-type *OdsH* over multiple generations. All allelic competition populations were studied for consecutive 20 generations, and population data were collected for generation numbers 1, 2, 3, 8, 12, and 20 (Fig. 6).

As shown in Fig. 6, the genotypic frequency of the wild-type *OdsH*(*w*) in adult males is recorded as $p_{1}$, which corresponds to the proportion of males with white eyes in the population; and the genotypic frequency of the homozygous wild-type *OdsH*/*OdsH*(*w*/*w*) in adult females is recorded as $Q_{11}$, which corresponds to the proportion of females with white eyes in the population. Genotypic frequencies were calculated by combining two replicate populations (statistics showed no significant differences between the replicates). The plots reflect the status of the wild-type *OdsH*(*w*) allele in direct competition, either with the ${OdsH}^{+}$ ($w^{+}$) allele (in red) or with the ${OdsH}^{1}$ ($w^{+}$) allele (in blue), during 20 generations of laboratory population setting. The eye-color *w* allows us to record the genotypic frequencies from the phenotypic frequencies of adult flies across the generations. However, it was difficult to distinguish between $w^{+}$/*w* (orange-red) and $w^{+}/w^{+}$ (dark-red) eye colors in females; therefore, it was most reliable to plot the genotypic frequencies for *w* (white eye) in males and *w*/*w* (white eye) in females.

It has been noted that *w* has pleiotropic effects on flies: *w* and *w*/*w melanogaster* flies have neurological deficiencies in addition to eye defects and likely suffer a fitness disadvantage (Ferreiro *et al.* 2018). Indeed, as shown in Fig. 6 (blue), we observed that genotypic frequencies decreased for both wild-type *OdsH*(*w*) males and wild-type *OdsH*/*OdsH*(*w*/*w*) females after 20 generations of direct competition with ${OdsH}^{+}$ ($w^{+}$) flies. Since *OdsH* and ${OdsH}^{+}$ both represent functional *OdsH* genes, fitness differences observed between *OdsH*(*w*) and ${OdsH}^{+}$ ($w^{+}$) can be attributed to the effects of genetic modification and the insertion of $w^{+}$. By contrast as shown in Fig. 6 (red), we observed that genotypic frequencies increased for both wild-type *OdsH*(*w*) males and wild-type *OdsH*/*OdsH*(*w*/*w*) females after 20 generations of direct competition with ${OdsH}^{1}$ ($w^{+}$) flies. Despite having a fitness advantage of $w^{+}$, ${OdsH}^{1}$ represents nonfunctional *OdsH*, resulting in an overall fitness disadvantage of ${OdsH}^{1}$ ($w^{+}$) flies competing directly against *OdsH*(*w*) flies. After 20 generations of laboratory population for allelic competition against a common reference, ${OdsH}^{1}$ was about 40% less frequent than ${OdsH}^{+}$, suggesting an overall fitness disadvantage from the loss of functional *OdsH*.

## Fitness component analysis

The fitness effect of the *OdsH* gene function is decomposed into individual viability and fertility components, independently, in males and females. It is important to separate viability and fertility effects and consider males and females separately because: (1) viability and fertility effects are often not correlated (Beckenbach 1983) and (2) a gene can have different functional effects in males and females, especially for an X-linked gene. *OdsH* is known to be active in the male testis of *D. melanogaster* and affect sperm production (Sun *et al.* 2004; Ting *et al.* 2004; Cheng *et al.* 2012). It is unknown whether the *OdsH*gene function would affect fly fitness in other ways.

In order to investigate fitness components associated with the gene function, we apply the “selection with two alleles of X-linkage” model on the experimental population data for two competing alleles of *OdsH*. We consider a single locus of X-linkage with two alleles $A_{1}$ and $A_{2}$:


$$\begin{array}{rl} A_{1} & \;:\;OdsH\;\text{(associated with}\;w);\\ A_{2} & \;:\;{OdsH}^{+}\;\text{(associated with}\;w^{+}). \end{array}$$

As described in the model, we use the following notations for adult frequencies in the population:


$$\begin{array}{rl} \;p_{1} & \;:\;\;\text{proportion of}\;A_{1}\;\text{males in the total males};\\ p_{2} & \;:\;\;\text{proportion of}\;A_{2}\;\text{males in the total males};\\ Q_{11} & \;:\;\;\text{proportion of}\;A_{1}A_{1}\;\;\text{females in the total females};\\ Q_{12} & \;:\;\;\text{proportion of}\;A_{1}A_{2}\;\;\text{females in the total females};\\ Q_{22} & \;:\;\;\text{proportion of}\;A_{2}A_{2}\;\;\text{females in the total females}. \end{array}$$

As described in the model, the ratios for genotypic frequencies and fitness components are expressed as:


$$\begin{array}{rl} \text{adult frequencies} & \;:\;R_{1}=\frac{\;p_{1}}{p_{2}},\;R_{2}=\frac{Q_{11}}{Q_{22}},\;R_{3}=\frac{Q_{22}}{Q_{12}};\\ \;\text{fertility effects} & \;:\;\alpha =\frac{a_{1}}{a_{2}},\;\beta =\frac{b_{11}}{b_{12}}=\frac{c_{11}}{c_{12}},\;\gamma =\frac{b_{22}}{b_{12}}=\frac{c_{22}}{c_{12}};\\ \text{viability effects} & \;:\;\mu =\frac{u_{1}}{u_{2}},\;\nu =\frac{v_{11}}{v_{12}},\;\epsilon =\frac{v_{22}}{v_{12}}. \end{array}$$

For generation *t*:


$$\begin{array}{rl} M_{t} & \;:\;\text{number of males in the population};\\ N_{t} & \;:\;\text{number of females in the population};\\ m_{t} & \;:\;\text{number of white-eyed}\;(w)\;\text{males in the population};\\ n_{t} & \;:\;\text{number of white-eyed}\;(w/w)\;\;\text{females in the population};\\ p_{1}^{\left(t\right)} & \;:\;\text{genotypic frequency of wild-type}\;OdsH\\ & \;(\text{represented by}\;w)\;\text{in adult males};\\ p_{2}^{\left(t\right)} & \;:\;\text{genotypic frequency of}{OdsH}^{+}\\ & \;(\text{represented by}\;w^{+})\;\text{in adult males};\\ Q_{11}^{\left(t\right)} & \;:\;\text{genotypic frequency of wild-type}\;OdsH/OdsH\\ & \;(\text{represented by}\;w/w)\;\text{in adult females};\\ Q_{12}^{\left(t\right)} & \;:\;\text{genotypic frequency of}\;{OdsH}^{+}/OdsH\\ & \;(\text{represented by}\;w^{+}/w)\;\text{in adult females};\\ Q_{22}^{\left(t\right)} & \;:\;\text{genotypic frequency of}\;{OdsH}^{+}/{OdsH}^{+}\\ & \;(\text{represented by}\;w^{+}/w^{+})\;\text{in adult females}. \end{array}$$

Two initial conditions (I, II) at generation 1:


$$\begin{array}{rl} \text{I} & \;:\;p_{1}^{\left(1\right)}=0,\;p_{2}^{\left(1\right)}=1,\;Q_{11}^{\left(1\right)}=0,\;Q_{12}^{\left(1\right)}=1,\;Q_{22}^{\left(1\right)}=0;\\ \text{II} & \;:\;p_{1}^{\left(1\right)}=1,\;p_{2}^{\left(1\right)}=0,\;Q_{11}^{\left(1\right)}=0,\;Q_{12}^{\left(1\right)}=1,\;Q_{22}^{\left(1\right)}=0. \end{array}$$

For ratios:


(4) $$\text{Setup I}\;:\;R_{1}^{\left(1\right)}=0,\;R_{2}^{\left(1\right)}=0,\;R_{3}^{\left(1\right)}=0,$$

(5) $$R_{1}^{\left(2\right)}=\mu ,\;R_{2}^{\left(2\right)}=0,\;R_{3}^{\left(2\right)}=\epsilon ,$$

(6) $$\text{Setup II}\;:\;R_{1}^{\left(1\right)}=\infty ,\;R_{2}^{\left(1\right)}=0,\;R_{3}^{\left(1\right)}=0,$$

(7) $$R_{1}^{\left(2\right)}=\mu ,\;R_{2}^{\left(2\right)}=\nu ,\;R_{3}^{\left(2\right)}=0.$$

For adult genotypic frequencies:


(8) $$p_{1}^{\left(t\right)}=\frac{R_{1}^{\left(t\right)}}{R_{1}^{\left(t\right)}+1},$$

(9) $$Q_{11}^{\left(t\right)}=\frac{R_{2}^{\left(t\right)}}{R_{2}^{\left(t\right)}+R_{3}^{\left(t\right)}+1}.$$

**Fig. 7.**
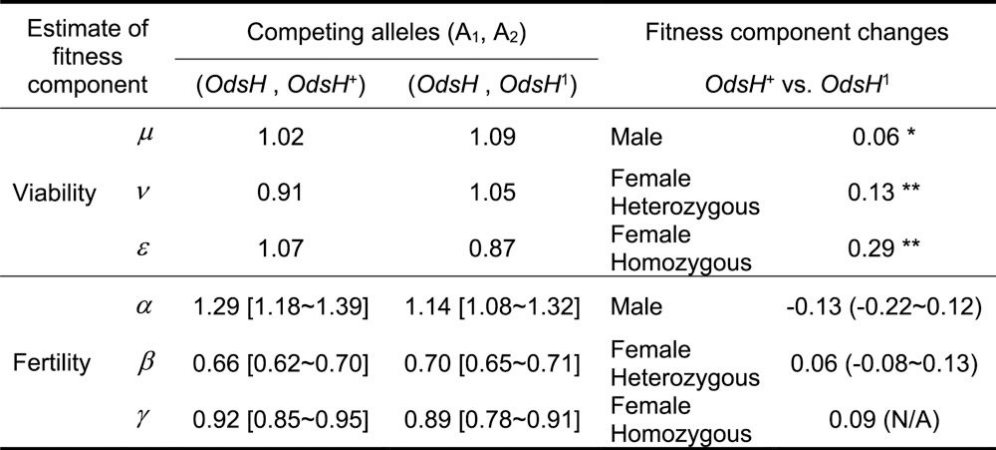
Estimates of fitness components for ${OdsH}^{+}$ and ${OdsH}^{1}$ as they each compete against the wild-type *OdsH* and derivations of fitness differences between ${OdsH}^{+}$ and ${OdsH}^{1}$. Frequency values are included in Supplementary Table S1. The estimates for *μ*, *ν*, and ϵ were first derived from $G_{2}$ according to equations (4) and (6) and were set as known ratios for viability effects. The three parameters for fertility effects, *μ* , *ν* , and ϵ, were estimated according to maximum likelihood criteria; confidence intervals (based on 95% $\chi^{2}$ value with one degree of freedom) for the fertility estimates are shown in the brackets next to the best-fit values. The fitness changes of ${OdsH}^{+}$ over ${OdsH}^{1}$ are calculated as follows: male viability increase $=1-\mu (OdsH,{OdsH}^{+})/\mu (OdsH,{OdsH}^{1}$ ); heterozygous female viability increase $=1-\nu (OdsH,{OdsH}^{+})/\nu (OdsH,{OdsH}^{1}$ ); homozygous female viability increase $=1-(\epsilon (OdsH,{OdsH}^{1})/\nu (OdsH,{OdsH}^{1}))/(\epsilon (OdsH,{OdsH}^{+})/\nu (OdsH,{OdsH}^{+}))$; male fertility increase $=1-\alpha (OdsH,{OdsH}^{+})/\alpha (OdsH,{OdsH}^{1})$; heterozygous female fertility increase $=1-\beta (OdsH,{OdsH}^{+})/\beta (OdsH,{OdsH}^{1})$; homozygous female fertility increase $=1-(\gamma (OdsH,{OdsH}^{1})/\beta (OdsH,{OdsH}^{1}))/(\gamma (OdsH,{OdsH}^{+})/\beta (OdsH,{OdsH}^{+}))$. Statistical significance for the viability changes is indicated: *P < 0.05 or **P < 0.001, with a $\chi^{2}$ test. The confidence intervals for the estimates of fertility changes are shown in the parentheses next to the values.

A computer program was written to compute the recursion relations (1), (2), and (3) with the initial conditions (4) and (6). To avoid the unrealistic computation load for six parameters, the estimates for *μ*, *ν*, and ϵ were first derived from $G_{2}$ according to (5) and (7) and were set as known ratios for viability effects (Fig. 7). The three parameters for fertility effects, *α*, *β*, and *γ*, were each taken to range from 0.1 to 10 with a log interval of 0.01, later on, being more specifically from 0.5 to 1.5 with an interval of 0.01. In fitting the experimental data with the relations (8) and (9), we took the maximum likelihood approach for the best estimates for parameters. The composite likelihood is written as follows:


$$\begin{array}{rl} L & =\prod_{t}\{\left[\frac{M_{t}!}{m_{t}!(M_{t}-m_{t})!}(p_{1}^{\left(t\right)}{)}^{m_{t}}(1-p_{1}^{\left(t\right)}{)}^{\left(M_{t}-m_{t}\right)}\right]\\ & \;\times \left[\frac{N_{t}!}{n_{t}!(N_{t}-n_{t})!}(Q_{11}^{\left(t\right)}{)}^{n_{t}}(1-Q_{11}^{\left(t\right)}{)}^{\left(N_{t}-n_{t}\right)}\right]\},\\ \ln\;L & =\text{Constant}+\sum_{t}\left[m_{t}\ln\;p_{1}^{\left(t\right)}+(M_{t}-m_{t})\ln\;(1-p_{1}^{\left(t\right)})\right]\\ & \;+\sum_{t}\left[n_{t}\ln\;Q_{11}^{\left(t\right)}+(N_{t}-n_{t})\ln\;(1-Q_{11}^{\left(t\right)})\right]. \end{array}$$

The combination of the three parameters (*α*, *β*, *γ*) estimates giving the largest lnL value was taken as the ratios for fertility effects. We use model fitting to find the best estimates of the three parameters based on maximum likelihood criteria. The estimates of *α*, *β*, *γ* infer the fertility effects as ratios for the two alleles in the male, in the heterozygous female, and in the homozygous female (Fig. 7).

As with *OdsH* and ${OdsH}^{+}$, we perform the same fitness component analysis for *OdsH* and ${OdsH}^{1}$ in parallel and determine the best estimates of the six parameters: *μ*, *ν*, and ϵ inferring viability effects, and *α*, *β*, and *γ* inferring fertility effects, as ratios for the two competing alleles ($A_{1}$ and $A_{2}$) (Fig. 7).

As shown in Fig. 7, we determine the effects of the *OdsH* gene function as fitness increases associated with ${OdsH}^{+}$ over ${OdsH}^{1}$ in six categories: viability in male, viability in female heterozygous, viability in female homozygous, fertility in male, fertility in female heterozygous, and fertility in female homozygous, with values calculated using fitness component estimates derived from allelic competition. While the functional *OdsH* increases the viability of males (by 0.06) and females (by 0.13 in heterozygous and 0.29 in homozygous), its effect on fertility is complex. The functional *OdsH* increases female fertility for both heterozygous (by 0.06) and homozygous (by 0.09), but it decreases male fertility as ${OdsH}^{+}$ is less fertile than ${OdsH}^{1}$ (by 0.13). The functional *OdsH* is associated with overall increased genotypic frequencies, which is consistent with previous findings that *OdsH* has been under positive selection (Ting *et al.* 1998). The reduction of male fertility contradicts previous observations that *OdsH* enhances sperm production in the testis (Sun *et al.* 2004; Cheng *et al.* 2012). There may be differences in outcomes of genetic effects depending on the laboratory setting: multi-generation competition in a laboratory population vs single-generation sperm exhaustion on individual males. Additionally, the fertility component analyzed in this study is related but not identical to sperm production alone. In the Discussion section, we also explain and discuss the possibility of inaccuracies in parameter estimation based on the theoretical model.

## Discussion

X-linked genes are of particular interest in human, mouse, and *Drosophila* genetics because some traits affected by X-linkage may be different for males and females, and X-linked recessives expressed in males can have more noticeable fitness effects. To formulate how allele frequencies change over time as a result of selection of X-linked genes, we present in this study a “Selection with Two Alleles of X-linkage” model. The model describes the structure of fitness attributes associated with X-linkage by distinguishing selection pressures on male hemizygous alleles, female heterozygous alleles, and female homozygous alleles. By applying the model to *OdsH*, a gene known for hybrid male sterility in *Drosophila*, we complement the theoretical framework with experimental data collected in laboratory populations over discrete non-overlapping generations. The rapid evolution and potential fitness advantage of *OdsH* have previously been reported, with the gene function identified in sperm production (Ting *et al.* 1998; Sun *et al.* 2004; Ting *et al.* 2004; Bayes and Malik 2009; Cheng *et al.* 2012; Nunes *et al.* 2023). However, assigning fitness determinants and phenotypic attributes remains an ongoing challenge. It is important to analyze fitness components of X-linked genes because these attributes are fundamental in determining the evolutionary dynamics of newly evolved alleles, and the dynamics of X-linked alleles can depend upon sexually dimorphic effects directly related to sex chromosomes. Our model and analysis of *OdsH* provide an example of examining fitness attributes associated with X-linked alleles’ frequency dynamics over generations. Strengths and weaknesses of this approach are discussed, as well as limitations related to the accuracy of fitness component estimation.

A major challenge in measuring fitness components has been comparing alleles with identical genetic backgrounds, as different genetic backgrounds would otherwise obscure or bias fitness effects (Clark and Bundgaard 1984). To resolve the issue, allele-specific manipulation of a gene provides an advantage. The “Gene Targeting” method in *Drosophila* is an site-directed genetic engineering approach, which utilizes the double-strand-break activated DNA repair system to facilitate homologous recombination at the target site. The targeted gene thus can be modified with custom designs on a donor DNA construct introduced by P-element transformation (Rong and Golic 2000, 2001). The genetically perturbed allele and its wild-type counterpart can be compared in the same genetic background for functional differences and fitness effects. $w^{+}$ serves as a visible phenotypic marker in complete linkage with the testing alleles, allowing direct competition with a common *w* reference in a laboratory population.

Our model for selection with two alleles of X-linkage is very general, since it combines the mating success and the fecundity for fertility estimates. Using the model, genotypic independent sex-ratios are assumed, fitness values are considered constant across generations, and generations are considered independent. To fit the experimental data, maximum likelihood estimations are used. The likelihood function is similar to that of Barclay and Fitz-Earle (1988). As part of the data analysis, the adult frequencies in generation 2 are used to estimate viability ratios. Evolutionary changes and random drift are neglected. In order to estimate fertility effects, viability ratios and adult frequencies in generation 3, 8, 12, and 20 are used.

The estimates may be inaccurate because of two factors: (1) similar frequency change trajectories may be generated by very different selective mechanisms (Beckenbach 1983). Our model fitting was performed based on six parameter estimates. To determine the three viability estimates, we used empirical values from generation 2, and the likelihood surface for the other three parameters is assumed to be flat. The low sensitivity of the fitting approach mostly affects the estimate of male fertility. Within the range of log likelihood value going from the maximum to a decrease of 1/2 of the 95% $\chi^{2}$ value (d.f.=1), it is possible to estimate male fertility ratios in the interval of 1.08–1.32 for the (*OdsH*, ${OdsH}^{1}$) competition and 1.18–1.39 for the (*OdsH*, ${OdsH}^{+}$) competition. Therefore, the fertility increase for ${OdsH}^{+}$ over ${OdsH}^{1}$ can be anywhere between − 0.22 and 0.12. (2) We did not consider frequency-dependent selection effects. This is particularly relevant with the idea of “minority-male mating advantage”, where Drosophila females mate preferentially with those males that are the rarest in the population; the effect generally increases with the rarity of the type of male (Ehrman and Parsons 1976; Hartl and Jungen 1979). Due to this, there is likely a frequency-dependent selection force that may affect the estimations of fitness effects, which we assumed to be constant across generations and between setup I and setup II. In spite of these potential inaccuracies in estimating the fitness components, the analysis here suggests that the functional *OdsH* gene provides a selective advantage in a competitive environment. For more accurate estimates, experiments for specific fitness effects are required.

Our fitness component estimates suggest that *OdsH* function increases female fertility and decreases male fertility. However, the confidence intervals for fertility differences (0.06, 95% confidence interval (CI) [−0.08, 0.13] for heterozygous females and −0.13, 95% CI [−0.22, 0.12] for hemizygous males) include zero, which indicates considerable variability. It is generally difficult to measure fitness from population data, and the results are usually highly variable (Prout 1969, 1971a, 1971b; Clark 1979; Wu 1983; Clark and Bundgaard 1984; Bastide *et al.* 2022). Despite the limitations of the model and the large variances in our estimates of *OdsH* fertility effects, we have demonstrated a possible approach to assign fitness determinants by integrating theoretical formulations with experimental data for an X-linked gene, allowing for distinction of male hemizygous, female heterozygous, and female homozygous alleles. The X-linked *OdsH* also has functional effects over multiple generations, which can be examined by analyzing the fitness attributes of *OdsH* alleles on males and females in continuous populations. The functional *OdsH* variant results in increased genotypic frequencies after 20 generations of allelic competition despite variable fitness effects in males and females (Fig. 6 and Supplementary Table S1). This is consistent with the selection of *OdsH* function in *D. melanogaster* (Ting *et al.* 1998; Sun *et al.* 2004).

We maintained our laboratory populations in 3/4 pint bottles instead of larger cages for allele competition, thus certain factors were not accounted for in our selection model or analysis. The limited space in bottles may restrict food resources and affect environment conditions for growth; this can influence development time and fly body size, both of which could affect mating success and frequency during competition (Houle and Rowe 2003). Our model is based on separate effects of viability and fertility in males and females but does not take into account differences during development. Although *OdsH* expression has been considered male-biased and restricted to the testis (Sun *et al.* 2004; Ting *et al.* 2004), *D. melanogaster* transcriptome analysis revealed low levels of *OdsH* during embryonic, larval, and pupal stages in both males and females (Brown *et al.* 2014). As a result, *OdsH* could influence the developmental process from egg hatching, larval growth, to fly eclosing. We estimate fitness components based on our model and multi-generation allelic competition data that *OdsH* function could enhance male and female viability, indicating that *OdsH* function may be involved in aspects of development and may be active during development. It is possible that there are differences in development time caused by the *OdsH* variants, which would influence estimation of the fertility effects of *OdsH* function in males and females. Further studies of the functional effects of *OdsH* in the development of males and females may shed further light on the effects of *OdsH* function on individual fitness components in *Drosophila*.

## Supplementary Material

jkae157_Supplementary_Data

## Data Availability

Fly strains and DNA constructs are available upon request. The authors affirm that all data necessary for confirming the conclusions of the article are present within the article and figures. Supplemental material available at G3 online.
